# Niacin Protects against Butyrate-Induced Apoptosis in Rumen Epithelial Cells

**DOI:** 10.1155/2019/2179738

**Published:** 2019-10-13

**Authors:** Dan Luo, Zhipeng Peng, Le Yang, Mingren Qu, Xiaowen Xiong, Lanjiao Xu, Xianghui Zhao, Ke Pan, Kehui Ouyang

**Affiliations:** ^1^Jiangxi Provincial Key Laboratory of Animal Nutrition, Jiangxi Agricultural University, Nanchang 330045, China; ^2^Menon Animal Nutrition Technology Co. Ltd., Shanghai 201800, China; ^3^Kaihua County Animal Husbandry and Veterinary Bureau, Quzhou 324000, China

## Abstract

The effects and underlying mechanisms of butyrate and butyrate+niacin on apoptosis in sheep rumen epithelial cells were investigated. Cells were exposed to butyrate (0–140 mM) for 6 h. A low concentration (20 mM) of butyrate increased cell viability and promoted growth whereas high concentrations (40–140 mM) inhibited proliferation. Cells were then cocultured with 120 mM butyrate and niacin (0–100 mM) for 6 h. Niacin addition attenuated butyrate-induced cellular damage and promoted proliferation at 20–80 mM; 40 mM presented the optimal effect. Higher concentrations (100 mM) of niacin resulted in low cell viability. Subsequent experiments confirmed that 120 mM butyrate increased intracellular reactive oxygen species (ROS) production and reduced the intracellular total antioxidant capacity (T-AOC) versus the untreated control. Compared with 120 mM butyrate, cotreatment with 40 mM niacin significantly reduced the intracellular ROS content and increased the intracellular T-AOC. Flow cytometry analysis revealed that 120 mM butyrate increased the proportion of apoptotic cells by 17.8% versus the untreated control, and 120 mM butyrate+40 mM niacin treatment reduced the proportion of apoptotic cells by 28.6% and 39.4% versus the untreated control and butyrate treatment, respectively. Treatment with 120 mM butyrate increased caspase-9 and p53 mRNA levels and decreased the expression of Bcl-2 and Bax, and the Bcl-2/Bax ratio versus the untreated control. Treatment with 120 mM butyrate+40 mM niacin downregulated the expression of caspase-3 and p53 and increased the expression of Bcl-2 and Bax versus butyrate treatment alone but had no effect on the Bcl-2/Bax ratio. Thus, high concentrations of butyrate may induce rumen epithelial cell apoptosis by increasing oxidative stress and inducing caspase-9 and p53 expression. Cotreatment with niacin regulates apoptosis-related gene expression by reducing intracellular ROS production and DNA damage and downregulating caspase-3 and p53 expressions to protect rumen epithelial cells against butyrate-induced apoptosis.

## 1. Introduction

Butyrate, a short-chain fatty acid (SCFA), serves as an important energy source for ruminants [1], which promotes the growth and fission of rumen epithelial cells, and increases the size and number of rumen papillae in calves. (daily intraruminal infusions of 500 g n-butyrate) [2]. However, increased butyrate concentrations in the rumen may decrease ruminal pH, putting cattle at a high risk of ruminal acidosis [3, 4], which causes exfoliation of ruminal epithelium cuticles and erosion of the rumen. Kristensen and Harmon [5] found that when butyrate absorption in the rumen exceeds the metabolic capacity of the ruminal epithelium, apoptosis of rumen epithelial cells was enhanced. Butyrate has been shown to induce apoptosis in some human cells, including tumor cells [6] and cancer cells [7]. Accumulating evidence suggests that butyrate can increase oxidative stress [8], including the overproduction of highly reactive oxygen species (ROS) [9] owing to the disturbed prooxidant/antioxidant balance. ROS should regulate the binding of p53 to DNA, thus promoting the transcriptional activity of p53 [10]. Stabilization of p53 activity leads to their increased intracellular levels and subsequently promotes apoptosis by enhancing the expression of proapoptotic proteins such as Bax and Noxa [11]. However, it is unclear whether butyrate can induce apoptosis of rumen epithelial cells in this way.

Niacin is a precursor of the coenzymes nicotinamide adenine dinucleotide (NAD) and nicotinamide adenine dinucleotide-phosphate (NADP), which play important roles in mitochondrial respiration and have redox functions during cellular metabolism. It is reported that the supplement ruminant diets with niacin were unnecessary, because niacin from feed and ruminal microbes synthetic may be sufficient to meet their production requirements [12]. But recent studies have indicated that for high-producing cattle, supplementation of high concentrated diets with 800 mg/kg niacin can reduce the risk of ruminal acidosis, enhance the production of ruminal microbial proteins, increase nutrient ruminal degradation, and improve the growth performance of cattle *in vivo* [13]. In addition, some researches have shown that niacin can prevent excessive apoptosis [14–16] and cell membrane damage. Li et al. [17] found that NAD^+^ can reduce intracellular phosphatidylserine (PS) ectropion and DNA damage. However, it remains unknown whether niacin can inhibit the apoptosis of rumen epithelial cells and help to alleviate the symptoms of rumen sedimentation caused by ruminal acidosis. Therefore, the objectives of this study were to investigate the effects and underlying mechanism of butyrate-induced rumen epithelial cell apoptosis and to explore the protective effects of niacin on rumen epithelial cells.

## 2. Materials and Methods

### 2.1. Reagents

Butyrate and niacin were obtained from Sigma, and Dulbecco's modified Eagle's medium (DMEM) was from Wisent. Phosphate-buffered saline (PBS), MTT, dimethyl sulfoxide (DMSO), amphotericin B, penicillin and streptomycin, 0.25% trypsin +0.02% EDTA, and 0.1% collagenase I were purchased from Solarbio. Epidermal growth factor (EGF) was obtained from Corning. ITS was purchased from ScienCell. Fetal bovine serum (FBS) was from BI, and ROS, T-AOC, and Annexin V-FITC/PI kits were obtained from Jiancheng.

### 2.2. Isolation and Culture of Rumen Epithelial Cells

This study was approved by the Animal Care and Use Committee of the College of Animal Science and Technology of Jiangxi Agricultural University.

Rumen epithelial cells were isolated from the rumen epithelial tissues of Hu sheep (aged 3–5 days, breastfeeding, both sexes). Immediately after death, the epithelium of sheep was quickly excised and the tissues were placed in ice-cold PBS. The tissues were repeatedly rinsed until the PBS remained clear. The rumen epithelial tissues were transported immediately to the laboratory. The following steps were performed in biohazard safety equipment. Briefly, the rumen epithelial tissues were washed five times with ice-cold PBS with 0.5 mg/mL amphotericin B and 100 *μ*g/mL gentamicin. The tissues were separated from the muscle layers, cut into small pieces (about 1 cm^3^), and washed twice with D-Hank's buffer. Subsequently, the rumen epithelial cells were isolated from the mucosae using 0.25% trypsin-0.02% EDTA and 0.1% collagenase I. Then, the rumen epithelial cells were seeded sequentially at a density of 1 × 10^6^ cell/mL and cultured in DMEM supplemented with 10% (*v*/*v*) FBS, 0.5% mEGF, 0.1% ITS, and 1% (*v*/*v*) streptomycin/amphotericin B in 25 cm^2^ plastic cell culture flasks at 37°C and 5% CO_2_ in an incubator (Thermo Fisher Scientific, Rockford, USA). The medium was replaced every 24 h, and the pH was maintained at 7.4.

To passage, rumen epithelial cells were detached using 0.25% trypsin-0.02% EDTA in PBS and then seeded at 3 × 10^5^ cells/25 cm^2^ in culture flasks at 37°C with 5% CO_2_. In the present study, we used rumen epithelial cells at passages 1–3; no immortal rumen epithelial cell line is available.

### 2.3. Cell Treatment

Rumen epithelial cells were seeded in 96- and 6-well plates in the appropriate medium. Experiments were performed when cells reached 80%. Cell culture media were maintained at pH 7.4. First, cells were exposed to different concentrations (0–140 mM) of butyrate for 6 h in 96-well plates with six repetitions per group, to determine the effect of butyrate on cell viability. Then, the cells were cocultured with an appropriate concentration (obtained from the first test) of butyrate and different concentrations (0–100 mM) of niacin for 6 h in 96-well plates with six repetitions per group, to determine the effect of niacin on cell viability. After that, the cells were cocultured with butyrate and niacin (optimum dose determined from the above tests) for 6 h in 6-well plates with three repetitions per group, to determine the ROS, total antioxidant capacity (T-AOC), cell apoptosis index, and the relative expression of apoptosis-related genes.

### 2.4. Cell Viability Assay

Cell viability was measured by MTT assay as previously described [18]. In brief, rumen epithelial cells were seeded in 96-well plates at a density of 5 × 10^3^/well and treated with butyrate and/or niacin at 37°C. After 6 h, 5 mg/mL MTT was added, and after 4 h further, MTT was replaced with 150 *μ*L DMSO/well. The cells were lysed by placing the plates on a shaker for 10 min to solubilize the formazan crystals within the cells. Absorbance at 490 nm was measured with a microplate spectrophotometer (Thermo Fisher Scientific, Rockford, USA). All independent experiments were performed in triplicate. Change in relative cell viability was presented as a response ratio (RR), which was calculated by the following formula:

(1) $$\begin{array}{c} \text{RR}=\ln\;\left(\frac{{\text{OD}}_{\text{treated}}}{{\text{OD}}_{\text{untreated}}}\right), \end{array}$$

where RR represents the response ratio, OD_treated_ indicates the OD value of treated cells, OD_untreated_ is the OD value of untreated cells, RR > 0 is an increase in viability in treated cells compared with untreated cells, and RR < 0 is a decrease in the viability of treated cells compared with untreated cells.

### 2.5. Determination of Intracellular ROS Level

Intracellular ROS levels were measured using 2′,7′-dichlorodihydro-fluorescein diacetate (DCFH-DA) following the manufacturer's protocol. Rumen epithelial cells were collected and washed twice with PBS. After centrifuging, cells were incubated with 10 *μ*M DCFH-DA for 30 min at 37°C in the dark. Subsequently, cells were washed twice and then resuspended in PBS. Fluorescence was measured using a plate reader (Thermo Fisher Scientific, Rockford, USA) with an excitation wavelength of 485 nm and an emission wavelength of 530 nm. The results were expressed as the fluorescence value.

### 2.6. Determination of Intracellular T-AOC

Intracellular T-AOC was estimated using a commercial kit according to the manufacturer's protocol. Cells were collected, centrifuged for 5 min at 1200 rpm, and resuspended in PBS. After cell disruption, homogeneous protein and T-AOC were measured at 595 and 520 nm, respectively, with a spectrophotometer (Thermo Fisher Scientific, Rockford, USA). T-AOC levels were expressed per mg of protein (U/mg prot).

### 2.7. Annexin V-FITC/PI Apoptotic Assay

Annexin V and propidium iodide (PI) staining were used to quantify the number of apoptotic cells. Rumen epithelial cells were collected and washed twice with PBS. After centrifuging, cells were resuspended in 500 *μ*L of binding buffer in a flow cytometric tube, to which 5 *μ*L of Annexin V-FITC and 5 *μ*L of PI were added and mixed well. After incubating for 10 min at room temperature in the dark, the stained cells were analyzed by flow cytometry (Becton Dickinson, San Jose, USA).

### 2.8. Real-Time Polymerase Chain Reaction

Total RNA was extracted from rumen epithelial cells with an RNA kit (Takara) according to the manufacturer's instructions. The amount and quality of RNA were determined using a spectrophotometer (Bio-Rad Laboratories, Hercules, USA). Total RNA (2.0 *μ*g) was then reverse transcribed to form cDNA using Transcript reverse transcriptase (Bio-Rad Laboratories, Hercules, USA) for quantitative real-time PCR, according to the manufacturer's instructions. Every reaction consisted of 2 *μ*L cDNA, 1 *μ*L of each primer (100 pmol), 1 *μ*L of dNTP Mix (0.5 mmol/L final concentration), and 9.5 *μ*L RNAse-free ddH_2_O (total reaction volume 14.5 *μ*L). Selected genes were quantified by RT-PCR using Primer 5.0 software according to the gene sequence provided by GenBank in NCBI. Primer sequences are shown in Table 1. RT-PCR cycles consisted of 95°C for 3 min, followed by 45 cycles of 95°C for 7 s, 57°C for 10 s, and 72°C for 15 s (Roche, Applied Science, Mannheim, Germany). Fold change in target gene expression was normalized by that of GAPDH using the 2^-*△△*Ct^ method.

### 2.9. Statistical Analysis

All results are presented as the mean ± standard deviation (SD). GraphPad Prism 5.01 software and Statistical Package for the Social Sciences (SPSS 17.0) packages were used for statistical analyses. Differences among groups were tested using a one-way analysis of variance (ANOVA). Duncan's multiple comparison test was used to compare statistical differences between treatments. A value of *P* < 0.05 was considered to indicate a significant difference.

## 3. Results

### 3.1. Effects of Butyrate on the Relative Viability of Rumen Epithelial Cells

**Figure 1:**
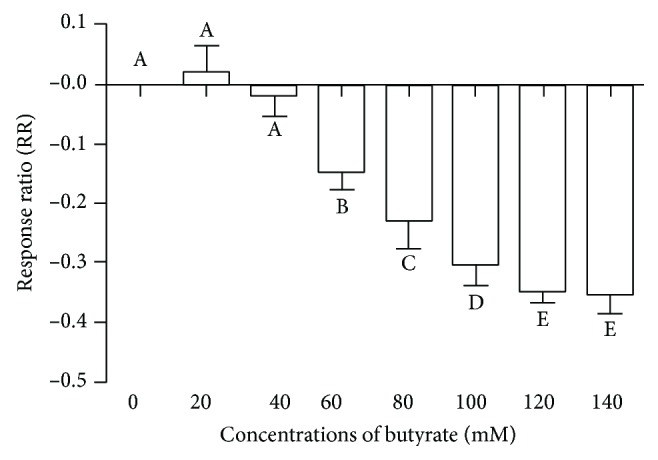
Effects of different concentrations of butyrate on the response ratio (RR) of relative cell viability. Rumen epithelial cells were treated with various concentrations of butyrate for 6 h, and cell viability was determined by MTT assay. Data are presented as the RR. RR = ln (OD_treated_/OD_untreated_), where RR is the response ratio, OD_treated_ refers to the OD value of treated cells, OD_untreated_ is the OD value of untreated cells, RR > 0 indicates an increase in relative viability in treated cells compared with untreated cells, and RR < 0 indicates a decrease in the relative viability of treated cells compared with untreated cells. Values with different letters indicate significant differences (*P* < 0.05).

First, we examined whether butyrate affects the relative viability of rumen epithelial cells. Data are presented as the RR as shown in Figure 1. The results showed that the relative viability was higher in cells treated with 20 mM butyrate compared with untreated cells, although this was not statistically significant (*P* > 0.05). However, cell growth was significantly inhibited following exposure to butyrate at 60–120 mM (*P* < 0.05), and the differences in relative cell viability were not significant following treatment with butyrate 120–140 mM (*P* > 0.05). Therefore, 120 mM butyrate was confirmed as the optimum dose for use in subsequent experiments.

### 3.2. Effects of Niacin on the Relative Viability of Rumen Epithelial Cells Cocultured with Butyrate

**Figure 2:**
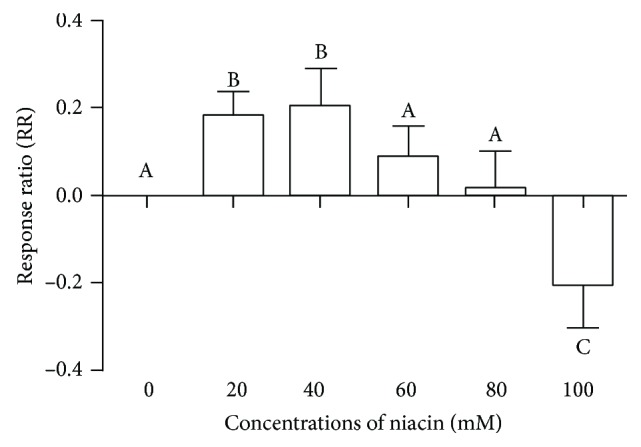
Effects of different concentrations of niacin on the response ratio (RR) of relative cell viability. Rumen epithelial cells were cotreated with 120 mM butyrate and various concentrations niacin for 6 h, and cell viability was determined by MTT assay. Data are presented as the RR. RR = ln (OD_(butyrate+niacin)-treated_/OD_butyrate-treated_), where RR is the response ratio, OD_butyrate-treated_ is the OD value of butyrate-treated cells, OD_(butyrate+niacin)-treated_ is the OD value of cells cotreated with butyrate and niacin, RR > 0 indicates an increase in the relative viability of cells cotreated with butyrate and niacin compared with butyrate-treated cells, and RR < 0 indicates a decrease in the relative viability of cells cotreated with butyrate and niacin compared with butyrate-treated cells. Values with different letters indicate significant differences (*P* < 0.05).

We further investigated the effect of niacin on butyrate-induced damage in rumen epithelial cells. The results are presented as the RR as shown in Figure 2. The results showed that, compared with butyrate treatment alone, 20 and 40 mM niacin significantly increased relative cell viability (*P* < 0.05), which peaked with 40 mM niacin. Niacin at 60 and 80 mM had no effect on relative cell viability (*P* > 0.05), while 100 mM significantly decreased relative cell viability (*P* < 0.05) and was found to be cytotoxic.

### 3.3. Effects of Butyrate and Niacin on Intracellular ROS Levels and T-AOC in Rumen Epithelial Cells

**Figure 3:**
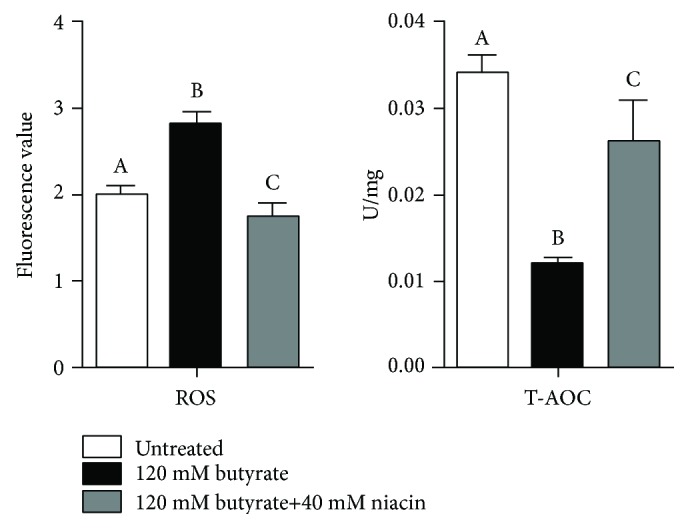
Effects of butyrate and niacin on the intracellular reactive oxygen species (ROS) and the intracellular total antioxidant capacity (T-AOC) of rumen epithelial cells. Rumen epithelial cells were exposed to 120 mM butyrate with or without 40 mM niacin for 6 h. Intracellular ROS levels were measured using 2′,7′-dichlorodihydro-fluorescein-diacetate (DCFH-DA). Intracellular T-AOC was estimated using a commercial kit according to the manufacturer's protocol. Data are presented as the mean ± standard deviation (SD). Values with different letters indicate significant differences (*P* < 0.05).

120 mM butyrate and 120 mM butyrate+40 mM niacin were used in subsequent experiments. As shown in Figure 3, intracellular ROS levels were significantly increased and intracellular T-AOC was significantly decreased in cells treated with butyrate alone (*P* < 0.05) compared with untreated cells. Cotreatment with niacin significantly attenuated the excess generation of intracellular ROS and enhanced the intracellular T-AOC in butyrate-stimulated cells (*P* < 0.05). Furthermore, it significantly decreased the intracellular ROS levels in untreated cells (*P* < 0.05). The intracellular T-AOC in untreated cells was not improved by the addition niacin and was lower than that observed in untreated cells (*P* < 0.05).

### 3.4. Effects of Butyrate and Niacin on the Apoptosis of Rumen Epithelial Cells

**Figure 4:**
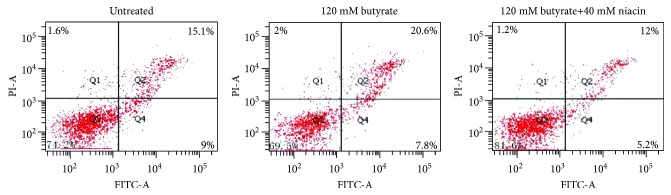
Apoptosis in rumen epithelial cell apoptosis determined by flow cytometry.

To determine whether cellular viability is affected by butyrate treatment, cells were analyzed by flow cytometry following Annexin-V FITC/PI double staining. As shown in Figure 4 and Table 2, butyrate treatment significantly decreased the proportion of normal cells and increased the proportion of late-apoptotic cells compared with the untreated control (*P* < 0.05). The rate of apoptosis with butyrate significantly increased by 17.8% compared with the untreated control (*P* < 0.05). Cotreatment with niacin markedly increased the proportion of normal cells and decreased the proportion of necrotic and late-apoptotic cells compared with butyrate treatment alone (*P* < 0.05). The rate of apoptosis following butyrate+niacin treatment was significantly decreased by 28.6% (*P* < 0.05) compared with the untreated control and was significantly decreased by 39.4% (*P* < 0.05) compared with butyrate treatment alone.

Rumen epithelial cells were exposed to 120 mM butyrate with or without 40 mM niacin for 6 h. Apoptosis was examined by Annexin V-FITC/PI double staining and analyzed by flow cytometry. Data are presented as the mean ± standard deviation (SD). Values with different letters indicate significant differences (*P* < 0.05).

### 3.5. Effects of Butyrate and Niacin on the Relative Expression of Apoptosis-Related Factors in Rumen Epithelial Cells

**Figure 5:**
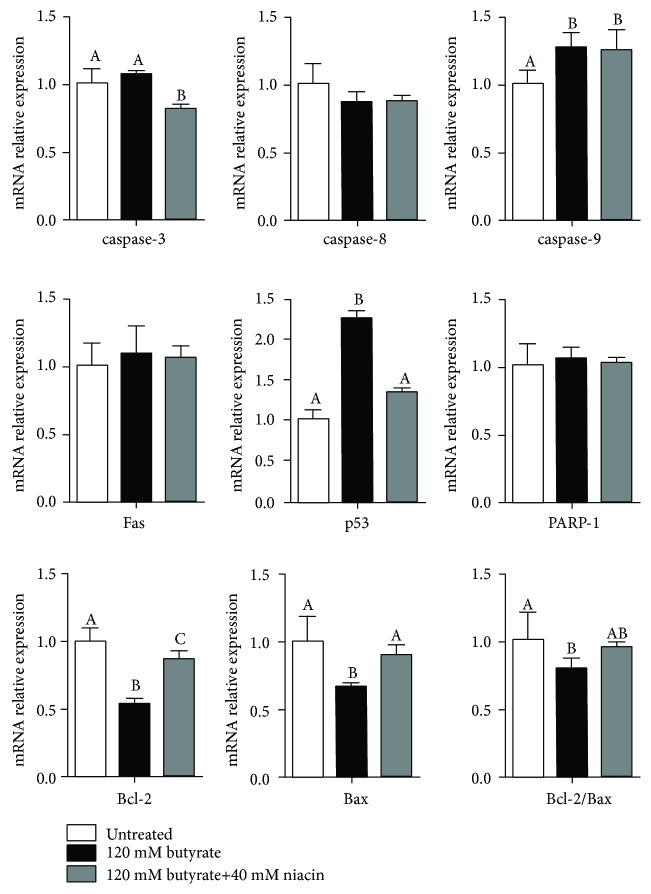
Effects of butyrate and niacin on the relative mRNA expression of apoptosis-related genes in rumen epithelial cells. Rumen epithelial cells were exposed to 120 mM butyrate with or without 40 mM niacin for 6 h. The expressions of caspase-3, caspase-8, caspase-9, Fas, p53, PARP-1, Bcl-2, and Bax mRNA were determined by RT-PCR analysis, and the ratio of Bcl-2/Bax was determined. Data are presented as the mean ± standard deviation (SD). Values with different letters indicate significant differences (*P* < 0.05).

As shown in Figure 5, compared with the untreated control, caspase-3 expression was not affected following butyrate treatment (*P* > 0.05); however, caspase-3 expression was significantly decreased in cells treated with butyrate+niacin (*P* < 0.05). Caspase-3 expression was lower with butyrate+niacin treatment than with butyrate treatment alone (*P* < 0.05). The expression of caspase-9 was significantly increased (*P* < 0.05) with butyrate treatment and butyrate+niacin treatment (*P* < 0.05) compared with the untreated control, but there were no significant differences between butyrate treatment and butyrate+niacin treatment (*P* > 0.05). P53 mRNA levels with butyrate treatment were higher than in the untreated cells and with butyrate+niacin treatment (*P* < 0.05); however, there were no significant differences between the untreated control and with butyrate+niacin treatment (*P* > 0.05). Butyrate treatment alone significantly decreased the expression of Bcl-2 and Bax and the ratio of Bcl-2/Bax compared with the untreated control (*P* < 0.05). Cotreatment with niacin markedly increased the expression of Bcl-2 and Bax (*P* < 0.05); however, there was no difference in the ratio of Bcl-2/Bax in butyrate-stimulated cells (*P* > 0.05). Butyrate exposure, with and without niacin, had no effect on caspase-8, Fas, and poly-ADP-ribose polymerase-1 (PARP-1) mRNA levels in rumen epithelial cells (*P* > 0.05).

## 4. Discussion

### 4.1. Effects of Butyrate and Niacin on the Growth of Rumen Epithelial Cells

Butyrate, a product of ruminal fermentation, is also an energy source for rumen epithelial cells [1]. The results of a previous study suggested that butyrate promotes cell proliferation by accelerating cell division [19] and inhibiting apoptosis [20]. In addition, butyrate can inhibit cell proliferation by stimulating apoptosis and promoting differentiation. The effects of butyrate on cell growth are cell- and concentration-dependent. Ruemmele et al. [21] found no differences in the viability of Caco-2 cells following treatment with 0.1–2 mmol/L butyrate for 48 h, whereas growth was inhibited with 100 mmol/L butyrate. Siavoshian et al. [22] reported that 1 mmol/L butyrate can hinder the growth of intestinal epithelial cells, while 8 mmol/L butyrate completely inhibited cell growth. In the current study, we found that a low concentration (20 mM) of butyrate promoted the growth of rumen epithelial cells, whereas high concentrations (40–140 mM) of butyrate inhibited cell proliferation. These results suggest that rumen epithelial cells may have tolerance to butyrate.

Niacin, also known as vitamin B_3_, is the dietary precursor for NAD(H) and NADP(H) synthesis. NAD participates in cellular metabolism and plays an important role in cellular proliferation and the repair of damaged cells [23]. Lin et al. [14] reported significant improvements in cell viability with a graded increase in niacin concentrations from 5 to 20 *μ*M, while 40 *μ*M niacin decreased viability of spontaneously immortalized human keratinocytes treated with ultraviolet irradiation. Liu et al. [24] showed that 200 mg/kg nicotinic acid or nicotinamide effectively increased the level of NAD^+^ and protected the functional cells in damaged brain tissue. The results of the present study indicated that low concentrations (20–80 mM) of niacin had a positive effect on the reduced cell viability induced by butyrate, similar to the results of previous studies. However, high concentrations (100 mM) of niacin decreased cell viability and presented toxic effects, whereby the observed proportion of dead cells may have been due to the duration of niacin exposure.

### 4.2. Effects of Butyrate and Niacin on the Redox State of Rumen Epithelial Cells

Overproduction of intracellular ROS can induce DNA and protein damage, which may contribute to cell death [9]. In this study, butyrate increased intracellular ROS production, decreased intracellular T-AOC, led to imbalanced redox levels in rumen epithelial cells, and increased cellular oxidative stress [25], which may be related to DNA double-strand DNA damage [26]. Niacin is an important antioxidant, which reduces oxidative damage through the conversion of NAD^+^ to NADP^+^ (the precursor for reductive NADPH formation) by NADK [27], the direct antioxidation effects of NADH [28], and reduced intracellular ROS production [29]. Here, we found that butyrate increased intracellular oxidative stress, which may induce apoptosis, as reported by Pant et al. [30] and Salimi et al. [31], and niacin can reduce butyrate-induced oxidative damage in cells; thus, these results may be related to the antioxidant function of niacin [32].

### 4.3. Effects of Butyrate and Niacin on the Apoptosis of Rumen Epithelial Cells

Our data indicate that high concentrations of butyrate inhibited the proliferation of rumen epithelial cells. Our results showed that the cytotoxic effect of butyrate was related to the induction of apoptosis. Sodium butyrate can also sensitize human pancreatic cancer cell lines via both the intrinsic and extrinsic apoptotic pathways, as reported by Natoni et al. [33]. In the present study, we demonstrated a significant accumulation of apoptotic cells during butyrate treatment. Meanwhile, cotreatment with niacin can prevent butyrate-induced apoptosis in rumen epithelial cells and decrease the proportion of apoptotic cells, as shown in a previous study [34]. This may be explained by the ability of NAD^+^ to reduce the ectropion of PS during prophase in apoptosis, DNA damage during anaphase in apoptosis [17], and promote ATP generation. In addition, Mateuszuk et al. [35] found that nicotinamide can deacetylate p53 proteins, directly inhibit p53 transcription and activation through sirt, and prevent growth arrest and inhibit apoptosis, thereby prolonging the life of cells *in vivo*. To further validate these findings, we determined the mRNA expression of genes associated with apoptosis. When cells are subjected to stress, apoptotic initiators, such as Fas, caspase-8, caspase-9, and Bax, are activated, followed by the executor caspase, caspase-3. Activated caspase-3 increases expression of PARP, which acts as a “DNA nick sensor” and plays an important role in the response to intracellular DNA damage [23]. P53 is an important transcription factor, which can participate in cell cycle progression and apoptosis by regulating the transcriptional activity of many genes [33]. In the present study, we found that compared with the untreated control, butyrate increased the expression of caspase-9 and p53 and decreased the expression of Bcl-2 and Bax and the ratio of Bcl-2/Bax. Therefore, we suggest that butyrate may induce apoptosis in rumen epithelial cells by increasing intracellular oxidative stress and inducing the expression of caspase-9 and p53. Meanwhile, compared with butyrate treatment alone, cotreatment with butyrate and niacin can decrease oxidative damage and the expression of caspase-3 and p53 and increase the expression of Bcl-2 and Bax. These results suggested that the inhibitory effect of niacin on butyrate-induced apoptosis in rumen epithelial cells may be related to improvement cell antioxidant capacity, promotion DNA repair, and the inhibition of downstream caspase-3 and p53 activation. However, expression of Fas, caspase-8, and PARP-1 was unchanged in rumen epithelial cells exposed to butyrate, regardless of the presence of niacin, indicating that butyrate-induced rumen epithelial cell apoptosis is independent of the extrinsic pathway, and the protective effect of niacin on butyrate-induced apoptosis does not occur through this pathway.

## 5. Conclusions

In summary, low concentrations of butyrate promoted the growth of rumen epithelial cells, whereas high concentrations induced apoptosis. The underlying mechanism may be related to butyrate increasing intracellular ROS levels and inducing the expression of caspase-9 and p53. Adding an appropriate concentration (40 mM) of niacin can inhibit butyrate-induced apoptosis of rumen epithelial cells, which may be associated with reduced intracellular oxidative stress, inhibition of caspase-3 and p53 activation, and DNA damage repair.

## Figures and Tables

**Table 1:** PCR amplification primer design.

Genes	Primer sequence	Origin	Size (bp)	Annealing temperature (°C)
GADPH	F: 5′-AGGTTGTCTCCTGCGACTTCA-3′	NM001190390.1	132	84.59
	R: 5′-CCCTGTTGCTGTAGCCGAAT-3′			
Fas	F: 5′-CTCTGAGGGGCTGAGATTGA-3′	NM001123003.1	107	82.44
	R: 5′-GTTTGCCAGGAGGACAAGG-3′			
Bcl-2	F: 5′-TGTTTGATTTCTCCTGGCTGT-3′	XM012103831	145	86.69
	R: 5′-ACTGCTTTCACGAACCTTTTG-3′			
Bax	F: 5′-TTCCGACGGCAACTTCAAC-3′	XM015100639.1	244	88.29
	R: 5′-GAGCACTCCAGCCACAAAGA-3′			
Caspase-3	F: 5′-GCAGCAAACCTCAGGGAAA-3′	XM15104559.1	154	80.98
	R: 5′-CATGGCTTAGAAGCACGCA-3′			
Caspase-9	F: 5′-TGTTGCCGTTTCCTTCTCC-3′	XM015099300.1	111	84.35
	R: 5′-CTAGCACTTCGCTTTCTGGTG-3′			
Caspase-8	F: 5′-AAAATGCCCTTCCCTTGTTG-3′	XM012142500.2	110	80.95
	R: 5′-CTTCCCTCTGTTCTGAGTCGGT-3′			
PARP-1	F: 5′-CTCCAATCGCTTCTACACCC-3′	XM_012118480.2	49	86.07
	R: 5′-AACCACCCCTGAGTAGACTGTAG-3′			
P53	F: 5′-CAGGAGACATTTTCCGACTTG-3′	NM001009403.1	122	83.89
	R:5′-TCATCCAGCCAGGTGACAA-3′			

**Table 2:** Effects of butyrate and niacin on rumen epithelial cell apoptosis (%).

	Normal cells	Necrotic cells	Early apoptotic cells	Late apoptotic cells	Apoptosis index
Untreated	74.3 ± 1.3^a^	1.6 ± 0.1^ab^	9.0 ± 1.1^a^	15.1 ± 1.6^a^	24.1 ± 1.3^a^
120 mM butyrate	69.6 ± 1.8^b^	2.0 ± 0.5^a^	7.8 ± 1.2^ab^	20.6 ± 1.8^b^	28.4 ± 2.3^b^
120 mM butyrate+40 mM niacin	81.6 ± 0.7^c^	1.2 ± 0.1^b^	5.2 ± 0.3^b^	12.0 ± 0.6^c^	17.2 ± 0.7^c^

## Data Availability

The data used to support the findings of this study are available from the corresponding author upon request.
